# Magnolol Modulates the RIG-I/NF-κB Signaling Pathway to Alleviate JEV Infection-Induced ST Cell Damage

**DOI:** 10.3390/ani16152389

**Published:** 2026-08-03

**Authors:** Bohan Zheng, Mengzhao Jiang, Hongjie Cui, Qinjin Li, Zhaoyan Lin, Xiaohong Huang

**Affiliations:** 1 College of Animal Science, Fujian Agriculture and Forestry University, Fuzhou 350002, China; 2 Key Laboratory of Traditional Chinese Veterinary Medicine and Animal Health in Fujian Province, Fuzhou 350002, China; 3 College of Marine Sciences, Fujian Agriculture and Forestry University, Fuzhou 350002, China

**Keywords:** Japanese encephalitis virus, ST cells, magnolol, nuclear factor-κB, inflammation, boar testicular inflammation

## Abstract

Japanese encephalitis virus (JEV) is a zoonotic pathogen. Infection in breeding boars triggers severe testicular inflammatory storms, resulting in testicular swelling, asthenospermia, oligozoospermia and even irreversible loss of reproductive capacity. Magnolol, a natural extract, exhibits remarkable anti-inflammatory and antioxidant activities. This study utilized swine testicular (ST) cells as an in vitro model to explore magnolol’s protective effects against JEV-mediated inflammation and its underlying mechanism. ST cells were infected with JEV at an MOI of 1 and treated with magnolol at 8, 12 and 16 μg/mL. We detected pro-inflammatory cytokine expression, performed 4D-FastDIA quantitative proteomics to screen differentially expressed proteins, verified key RIG-I and NF-κB cascade molecules at mRNA and protein levels, and tracked p65 nuclear translocation through immunofluorescence. The data showed that JEV significantly elevated TNF-α, IL-1β, IL-6, IL-8 and CCL5, while magnolol reduced these cytokines in a dose-dependent manner. JEV reshaped the proteome of innate immunity and inflammation, and excessively activated the RIG-I/NF-κB axis to increase IKKβ, TRAF2, TRAF6 transcription and p65 phosphorylation and nuclear import; 24 h treatment with 16 μg/mL magnolol greatly restrained this overactivation. In summary, magnolol alleviates JEV-triggered inflammatory injury by inhibiting the RIG-I/NF-κB pathway, offering experimental evidence for its use as a natural agent to mitigate JEV-induced cellular inflammatory response in boars.

## Simple Summary

Japanese encephalitis virus (JEV) represents a significant zoonotic infectious disease. Infection of boars with JEV can result in orchitis, with excessive inflammatory responses leading to testicular swelling, asthenospermia, and oligospermia, which may severely compromise the breeding value of boars. Magnolol, a polyphenolic biphenyl compound extracted from Magnolia officinalis, demonstrates pharmacological effects, including anti-inflammatory, antioxidant, antibacterial, and anti-tumor properties. This study indicates that magnolol can mitigate inflammation induced by JEV infection through the regulation of the RIG-I/NF-κB signaling pathway, thereby safeguarding ST cells.

## 1. Introduction

JEV belongs to the genus Flavivirus within the family Flaviviridae [1]. The viral genome is approximately 11 kb in length and contains a long open reading frame (ORF) that encodes three structural proteins and seven non-structural proteins. The 5′ and 3′ ends of the viral genome are flanked by two non-coding regions (NCRs) [2]. The JEV virion measures approximately 50 nm in diameter and consists of viral RNA and nucleocapsid proteins. Japanese encephalitis (JE), caused by JEV infection, is one of the most significant viral encephalitides globally, with approximately 50,000 annual infections [3]. JEV was first identified in Japan, and currently, approximately 25 countries or regions face the threat of Japanese encephalitis outbreaks. The affected areas extend from the China–Russia border in the north to northern Australia in the south, and from the western Pacific islands in the east to the India–Pakistan border in the west [4,5]. Mosquitoes serve as the transmission vectors for JEV [6], with the distribution of Culex tritaeniorhynchus overlapping significantly with the epidemiological regions of Japanese encephalitis, which is crucial for the spread of JEV [7].

Pigs serve as reservoir hosts for JEV and play a crucial role in the virus transmission cycle. After JEV infects pigs, the virus amplifies within their bodies, leading to viremia [8]. When mosquitoes bite infected pigs, the virus is transmitted to humans through these mosquitoes [9]. The tonsils of pigs play a significant role in JEV transmission. Studies have shown that after pigs are infected with JEV, the viral load in the tonsils is 100 to 1000 times higher than that in other organs, and JEV can still be detected in the tonsils up to 11 days after the end of viremia [10]. JEV can spread directly among pig populations through oral and nasal infections. In regions where JEV is endemic, the positivity rate in pig populations is relatively high. For instance, in pig farms in Taiwan, the positivity rate of JEV exceeds 50% [11], while in four provinces of northern Laos, the positivity rate of JEV ranges from 59% to 90% [12]. JEV can cause reproductive disorders in pigs, leading to significant economic losses in the breeding industry. Clinical symptoms include abortion in pregnant sows and orchitis in boars. When orchitis occurs in boars, symptoms such as edema and congestion of the testes, hardening of the epididymis, and decreased libido can be observed. Additionally, the total sperm count in affected boars decreases, sperm motility is reduced, and there is a significant presence of abnormal sperm. Following JEV infection, the size of the boar’s testes can be approximately 1.5 to 2 times the normal volume, and the testes become warm and fluctuate upon palpation. A few days later, the testes gradually harden and are atrophied, and the swelling subsides. Severely affected boars may suffer from permanent infertility even after the inflammation of the testicles has resolved. However, research on the specific mechanisms and treatment methods for orchitis caused by JEV infection in boars remains limited.

During the replication process, JEV generates a significant amount of viral RNA and proteins. The host recognizes pathogen-associated molecular patterns (PAMPs) through pattern recognition receptors (PRRs), which subsequently recruit adaptor proteins and transcription factors to express interferons and inflammatory mediators. These responses encompass the generation of inflammation and immune stress, among other immune reactions, to combat viral infection [13]. In the host’s defense against pathogen invasion, the RIG-I-MAVS-mediated antiviral response plays a crucial role. RIG-I-MAVS can continuously activate downstream transcription factors and interferon regulatory factors to induce immune responses. Previous studies have demonstrated that the pattern recognition receptor RIG-I can induce orchitis in boars infected with JEV by regulating the NF-κB signaling pathway [14]. However, it remains unclear whether inhibiting NF-κB can reduce the incidence of orchitis. The NF-κB signaling pathway is present in nearly all cell types, where it participates in physiological processes by regulating gene transcription [15], including inflammatory responses, cell division and growth, as well as apoptosis [16]. NF-κB can be activated through various mechanisms; for instance, intracellular TLR3 recognizes double-stranded RNA [17,18], while TLR7 and TLR8 can identify single-stranded RNA within cells and activate NF-κB [19]. Following the activation of pattern recognition receptors in dendritic cells or macrophages, the release of inflammatory mediators is dependent on NF-κB. The transcriptional activity of NF-κB can induce the release of inflammatory factors, which in turn can further activate NF-κB, exacerbating inflammation [20]. Therefore, further research is warranted to explore the inhibition of JEV-induced orchitis in boars by regulating the NF-κB signaling pathway.

Natural compounds have the advantages of low toxicity, high biosafety and multi-target pharmacological properties, making them promising antiviral candidates. They exert anti-JEV effects primarily by regulating host innate immune and inflammatory signaling pathways to suppress viral replication and relieve virus-triggered inflammatory damage. Consistent with this mechanism, existing studies have confirmed that natural active monomers such as stigmasterol and curcumin exhibit prominent anti-JEV regulatory potential [21,22]. Magnolol is an active monomer extracted from the natural product Magnolia officinalis, characterized by the molecular formula C_18_H_18_O_2_. This monomer appears as a colorless, needle-like crystal, exhibiting solubility in organic solvents while being poorly soluble in water. Magnolol demonstrates a broad spectrum of biological activities, including antioxidant [23], anti-inflammatory [24], enzyme regulatory activity, antiviral property, promotion of apoptosis, and anti-tumor effects [25]. As a polyphenolic compound, magnolol possesses notable anti-inflammatory and antioxidant properties. Previous studies have indicated that magnolol can modulate the NF-κB signaling pathway. For example, in human osteosarcoma cells, magnolol reduces the expression of anti-apoptotic and metastasis-related genes by inhibiting the ERK/NF-κB signaling pathway [26]. In the context of multiple myeloma, magnolol suppresses the NF-κB signaling pathway by upregulating microRNA-129, which inhibits cell migration and invasion while inducing apoptosis [27]. Furthermore, previous research has demonstrated that JEV infection triggers inflammation through the activation of the NF-κB signaling pathway [14], and magnolol exhibits the capacity to inhibit NF-κB. This project aims to conduct an in-depth analysis of the regulatory mechanisms of NF-κB in JEV-induced inflammation in male porcine testes and to investigate the intervention effects and pathways of magnolol. This theoretical advancement may provide scientific support for the development of targeted immune regulation treatment strategies, while also unveiling new mechanisms of action for antiviral therapy in immune regulation.

## 2. Materials and Methods

### 2.1. Cell Viability Assay

The ST cells were cultured in DMEM (Gibco, Invitrogen, Carlsbad, CA, USA) supplemented with 10% fetal bovine serum (FBS, Gibco, Invitrogen, Carlsbad, CA, USA) and 1% penicillin-streptomycin solution (100×, Gibco, Invitrogen, Carlsbad, CA, USA). They were maintained at 37 °C in a humidified incubator with 5% CO_2_. 

Cell viability was assessed using the conventional CCK-8 assay. Logarithmic-phase ST cells were seeded into 96-well plates at a density of 1 × 10^4^ cells per well. Ten milligrams of magnolol was dissolved in 100 μL DMSO and filtered through a 0.22 μm filter to prepare a magnolol stock solution at a concentration of 100 μg/μL. Cells were treated with gradient concentrations of magnolol (0, 4, 8, 12, 16, 20, 24, 28, and 32 μg/mL) diluted with DMSO. The final DMSO concentration was strictly maintained at 0.032% (*v*/*v*) in all cell treatment groups. Each concentration was tested with six replicates, and both cell control and blank control groups were established simultaneously. The plates were subsequently placed in a cell culture incubator for 24 h. After incubation, the 96-well cell culture plates were removed, and the medium in the cell wells was washed away in a clean bench environment. Each well was then filled with 100 μL of medium containing 10 μL of CCK-8 solution, and the plates were returned to the incubator for 3 h of light-protected cultivation. Following this period, the 96-well plates were removed, and the optical density (OD) values at 450 nm absorbance were measured using a microplate reader. Cell viability was calculated using the following formula based on the OD values: [(A control − A experimental)/(A control − A blank)] × 100%.

### 2.2. Quantitative Real-Time PCR (qPCR) Assay

The qPCR method was performed as follows: Treated cells were washed with PBS buffer, and total RNA was extracted using the Mini BEST Universal RNA Extraction Kit (Takara, Kusatsu, Shiga, Japan). Complementary DNA (cDNA) was synthesized via reverse transcription using the TaKaRa PrimeScript™ RT Master Mix (Perfect Real Time). Quantitative PCR (qPCR) was conducted on an ABI real-time fluorescence quantitative PCR instrument (Thermo Fisher Scientific, Waltham, MA, USA) using Takara TB Green^®^ Premix Ex Taq™ II (Tli RNaseH Plus). Gene-specific primers were purchased from Sangon Biotech, Shanghai, China. (Table 1). The qPCR experiment was repeated three times, with each gene having three technical replicates. The thermal cycling conditions for qPCR were as follows: initial denaturation at 95 °C for 30 s, followed by 40 cycles of denaturation at 95 °C for 5 s and annealing/extension at 60 °C for 30 s. A melting curve analysis was performed to confirm the specificity of the amplification products, which included a heat treatment at 95 °C for 5 s, incubation at 60 °C for 60 s, denaturation at 95 °C, and final cooling at 50 °C for 30 s. The relative mRNA expression levels of the target genes were calculated using the 2-ΔΔCt method, normalized to the housekeeping gene β-actin.

### 2.3. The Amplification of JEV

Spread BHK cells in a good growth state into T175 cell culture flasks. When the confluence of BHK cells reaches 80% to 90%, remove the flasks, discard the culture medium, and rinse the cells three times with serum-free DMEM. Subsequently, add serum-free DMEM containing 10^5^ viruses to the cell culture flasks and incubate them in a cell culture incubator. After 1.5 h, remove the flasks, discard the culture medium, and gently rinse the cells three times with PBS. Then, add an appropriate amount of cell maintenance fluid and return the flasks to the incubator for further cultivation. Once significant cytopathic effects, such as rounding, shrinkage, and detachment, are observed in the BHK cells, quickly place the T175 cell culture flasks in a −80 °C freezer for rapid freezing. After approximately 1 h, remove the flasks for thawing, gently shaking them during the process. Repeat this freezing and thawing cycle three times. Next, transfer the resulting liquid to a 50 mL centrifuge tube, ensuring the centrifuge has been pre-cooled to 4 °C. Place the 50 mL centrifuge tube in the centrifuge and centrifuge at 5500 rpm for 30 min. After centrifugation, transfer the supernatant to a new 50 mL centrifuge tube and filter it through a 0.22 μm filter, gently shaking to mix. Finally, dispense the filtered virus solution into 1.5 mL centrifuge tubes, label them appropriately, and store them in a −80 °C freezer.

### 2.4. The Effect of Magnolol on the Expression of Inflammatory Factors in JEV-Induced ST Cells

ST cells in optimal growth conditions were prepared into a cell suspension, with approximately 10^5^ cells per well seeded into a 12-well cell culture plate, which was subsequently placed in a cell culture incubator for cultivation. When the cells reached 80–90% confluency, the medium was aspirated, and the cells were washed three times with serum-free DMEM. Following this, serum-free DMEM containing 1 MOI of virus was added, and the plate was incubated in the cell culture incubator for 1.5 h. The cell culture plate was then removed and washed three times with PBS. The experiment was organized into five groups, each with three replicates: the blank group, the JEV group, and the JEV + Magnolol (8, 12, 16 µg/mL) groups. Magnolol was diluted with cell maintenance medium to concentrations of 8, 12, and 16 µg/mL. In the blank group, cell maintenance medium containing an equivalent volume of DMSO as in the JEV + Magnolol (16 µg/mL) group was added, and in the JEV group, cell maintenance medium containing the same volume of DMSO was used. Subsequently, the cell culture plate was placed back in the cell culture incubator and cultured for 24 h before collecting the total RNA from the cells, with the process being repeated three times.

### 2.5. D-FastDIA Quantitative Proteomics Assay

ST cells in optimal growth conditions were counted and evenly distributed into 12 T75 cell culture flasks. Once the cells reached 80% to 90% confluence, the culture medium was discarded, and the cells were washed three times with serum-free DMEM. The cells were then incubated in serum-free DMEM for 1.5 h. The JEV group and the JEV + Magnolol group were supplemented with 1 MOI of virus solution. After incubation, the DMEM was completely aspirated using an electric pipette. The Magnolol group and the JEV + Magnolol group were subsequently supplemented with 15 mL of cell maintenance medium containing 16 µg/mL Magnolol, while the control group and the JEV group received an equal volume of cell maintenance medium. It is essential to ensure that each group contains the same volume of DMSO, with three T75 flasks per group serving as replicates, and to place them in a cell culture incubator for 24 h. After 24 h, the 12 T75 cell culture flasks were removed and gently rinsed three times with PBS pre-cooled to 4 °C. A cell scraper was then used to gently detach the cells from the flasks, and the cells were observed under a microscope to confirm complete removal. The scraped cells were transferred to a centrifuge tube and centrifuged at 1000 rpm for 10 min. Following centrifugation, pre-cooled PBS was added to the centrifuge tube, and the mixture was gently pipetted to ensure homogeneity, followed by a second centrifugation. All PBS was then removed from the centrifuge tube. The centrifuge tubes containing the cell pellets were labeled and quickly frozen in liquid nitrogen for 2 min before being stored in a −80 °C freezer. Each set of protein quantifications was performed in duplicate, followed by digestion with trypsin. After digestion was completed, the sample volumes were normalized, and liquid chromatography-mass spectrometry (LC-MS) analysis was conducted. The obtained data were used for subsequent analysis.

### 2.6. D-FastDIA Quantitative Proteomics Analysis

1. Conduct a database search on the acquired data, encompassing the protein database, protein annotation, and DIA-NN library search. 2. Perform quality control analysis on the obtained data prior to data analysis, which includes assessing peptide length distribution and peptide number distribution. 3. Following the successful completion of quality control, execute quantitative analysis on the sample proteins, primarily focusing on quantitative distribution statistics, PCA, RSD analysis, Pearson correlation analysis, and intensity value distribution. 4. Carry out basic functional annotation of the proteins identified through the library search, which includes KEGG pathway functional annotation, Gene Ontology (GO) functional annotation, domain functional annotation, and COG/KOG functional annotation. 5. Compare the acquired protein data between groups, calculating based on T-test and *p*-value, and screening according to a 1.5-fold difference. 6. Conduct functional classification of the differential proteins between groups, which includes GO secondary classification, KEGG pathway statistics, COG/KOG classification, and perform enrichment analysis of the differential proteins between groups using Fisher’s exact test, encompassing GO, WikiPathways, KEGG, protein domain, and Reactome.

### 2.7. Western Blot

Protein samples were prepared with protease inhibitors (Beyotime, Shanghai, China) and RIPA lysis buffer (Beyotime, Shanghai, China). Protein concentration was measured by Bradford reagent (Sigma-Aldrich, St. Louis, MO, USA). A total of 20 μg of protein was loaded onto SDS-PAGE (8–12%) and transferred to PVDF membranes (Millipore, Billerica, MA, USA). Next, the membrane was blocked with 5% skim milk and incubated with the appropriate first antibody and the corresponding second antibody. ECL reagent (Advansta, Menlo Park, CA, USA) revealed protein bands.

### 2.8. Statistical Analysis

One-way analysis of variance (one-way ANOVA) was used to analyze experimental data. All results were presented as mean ± standard deviation. *p* < 0.05 was defined as statistically significant.

## 3. Results

### 3.1. Effects of Magnolol on the Cytotoxicity of ST Cells

**Figure 1:**
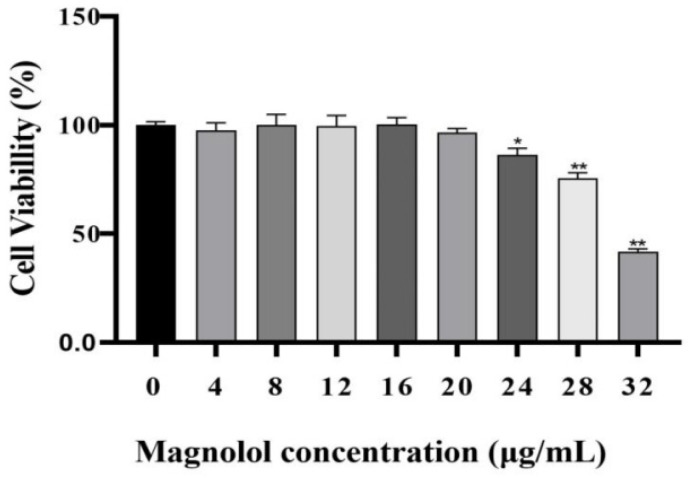
Effect of magnolol on ST cell viability (* *p* < 0.05; ** *p* < 0.01).

The CCK-8 colorimetric assay was adopted to evaluate the cytotoxicity of magnolol against swine testicular (ST) cells, quantify its influence on cellular viability and proliferation, and screen safe, non-toxic working concentrations for subsequent co-treatment experiments combined with JEV infection, with all detection results summarized in Figure 1. After ST cells were incubated with serially diluted magnolol for 24 h, obvious dose-dependent cytotoxicity was observed at relatively high doses. A moderate decline in cell viability was first detected at 20 μg/mL magnolol, while concentrations equal to or higher than 24 μg/mL triggered a statistically significant reduction in cell survival rate, indicating severe toxic damage to ST cells at these high magnolol doses. To eliminate the interference of magnolol-induced intrinsic cell death on the detection of JEV-triggered inflammatory responses and pathway activation, we excluded cytotoxic high concentrations and selected three gradient concentrations without obvious cellular toxicity, namely 8, 12 and 16 μg/mL magnolol, for all subsequent viral infection, qPCR and Western blot validation assays.

### 3.2. Effects of Magnolol on the Expression of Inflammatory Cytokines in JEV-Induced ST Cells

**Figure 2:**
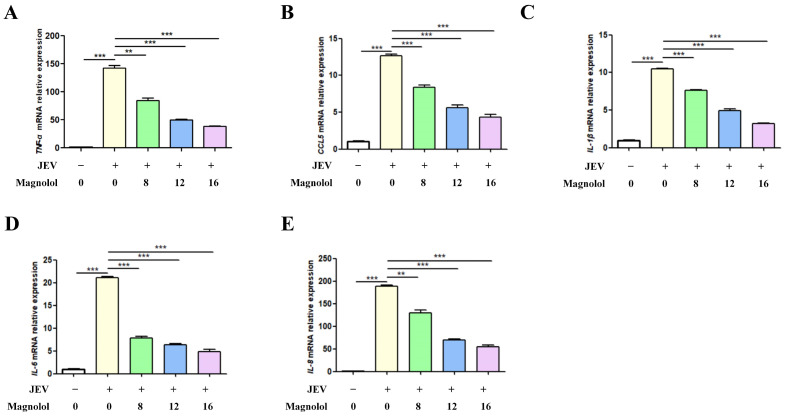
Effect of magnolol on expression of ST inflammatory factors induced by JEV; (**A**) Relative mRNA expression of *TNF-α* detected by qRT-PCR. (**B**) Relative mRNA expression of *CCL5* detected by qRT-PCR. (**C**) Relative mRNA expression of *IL-1β* detected by qRT-PCR. (**D**) Relative mRNA expression of *IL-6* detected by qRT-PCR. (**E**) Relative mRNA expression of *IL-8* detected by qRT-PCR. ** *p* < 0.01, *** *p* < 0.001. (*n* = 3 for each group).

To investigate the effects of magnolol at different concentrations on the expression of inflammatory cytokines in JEV-stimulated ST cells, five experimental groups were set up, including the control group and four JEV + magnolol groups treated with 0, 8, 12, and 16 μg/mL magnolol, respectively, followed by the detection of the expression levels of inflammatory cytokines. As shown in Figure 2, the mRNA transcription levels of *TNF-α*, *IL-1β*, *IL-6*, *IL-8* and chemokine *CCL5* were extremely significantly elevated in ST cells after JEV infection. Compared with the JEV single infection group, treatment with magnolol at all tested concentrations significantly suppressed the transcription of *TNF-α*, *IL-1β*, *IL-6*, *IL-8* and *CCL5*, and the expression abundance of these inflammatory cytokines decreased gradually with the increase in magnolol concentration.

### 3.3. Quantitative Proteomic Results Based on 4D-FastDIA

To explore the mechanism underlying the protective effect of magnolol against JEV-induced ST cell damage, ST cells were infected with JEV at 1 MOI. After 1.5 h of incubation, the cells were washed three times with DMEM. Cells were then cultured in maintenance medium containing 16 μg/mL magnolol or an equal volume of DMSO. After 24 h of incubation, 12 protein samples from four groups (Control, Magnolol, JEV + Magnolol, and JEV) were collected for 4D-FastDIA quantitative proteomic analysis.

All DIA data were searched using DIA-NN (V1.8) with default parameters. The Sus scrofa protein database (Sus-scrofa-9823-PR-20230529.fasta, 46,179 sequences) was used for protein identification. Trypsin/P was set as the digestion enzyme with a maximum of one missed cleavage. Fixed modifications included N-terminal methionine excision and cysteine carbamidomethylation. A deep learning-based theoretical spectral library and decoy database were used to calculate the false discovery rate (FDR), with the identification FDR threshold set to 1%.

**Figure 3:**
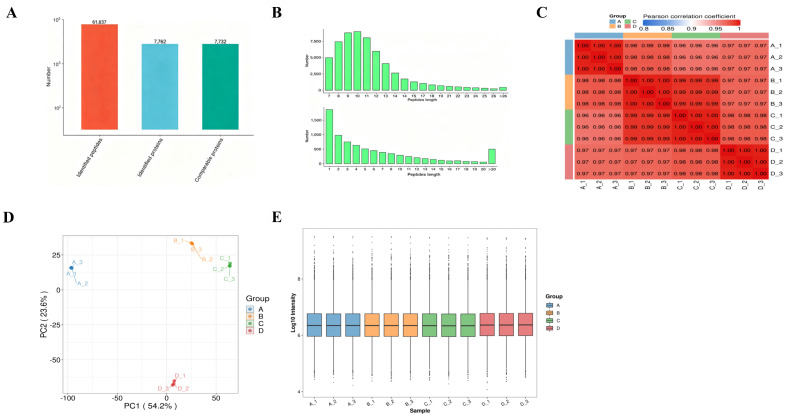
Results of 4D-FastDIA quantitative proteomic analysis. (**A**) Statistics of identified peptides and proteins. (**B**) Length and abundance distribution of identified peptides. (**C**) Pearson correlation heatmap for 12 protein samples across four groups. (**D**) Principal component analysis (PCA) of the 12 protein samples. (**E**) Relative standard deviation (RSD) of the 12 protein samples.

Data were further filtered with a 1% protein-level FDR and the requirement of at least one unique peptide. A total of 61,837 peptides were identified, corresponding to 7762 proteins with unique peptides, among which 7732 proteins were quantitatively analyzable (Figure 3A). Peptide length distribution and matching numbers were evaluated for quality control. Most identified peptides ranged from eight to 11 amino acids in length, and most proteins possessed more than two unique peptides, satisfying the mass spectrometry quality requirements (Figure 3B).

Pearson correlation analysis was performed on 12 biological replicate samples to verify experimental reproducibility. The correlation coefficients among samples were all above 0.96, indicating excellent intra-group consistency (Figure 3C). Principal component analysis (PCA) based on protein quantitative values showed that samples from the same group clustered closely, demonstrating good repeatability and reliable grouping (Figure 3D). Boxplots of relative standard deviation (RSD) for the four groups further confirmed the stable quantitative results of the biological replicates (Figure 3E).

### 3.4. Screening of Differentially Expressed Proteins

**Figure 4:**
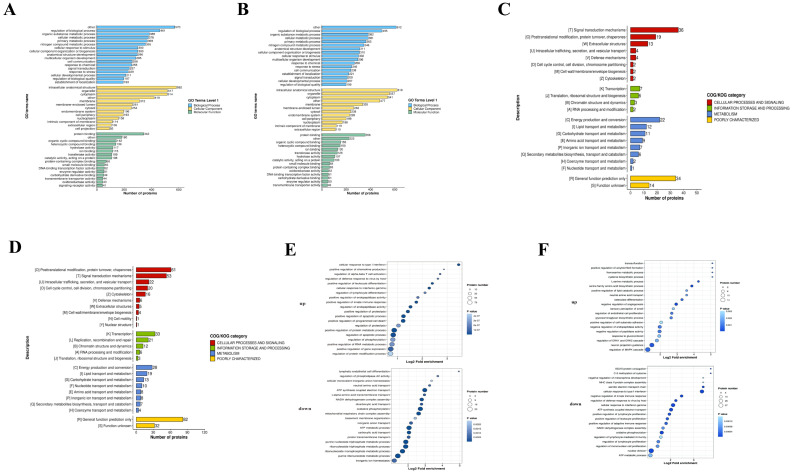
Screening of differentially expressed proteins. (**A**) GO secondary classification of differentially expressed proteins in the D/A comparison group; (**B**) GO secondary classification of differentially expressed proteins in the C/D comparison group; (**C**) COG/KOG functional classification of differentially expressed proteins in the D/A group; (**D**) COG/KOG functional classification of differentially expressed proteins in the C/D group; (**E**) bubble plot of KEGG pathway enrichment of differentially expressed proteins in the D/A group; (**F**) bubble plot of KEGG pathway enrichment of differentially expressed proteins in the C/D group.

Gene Ontology (GO) secondary classification annotates protein properties from three core dimensions: cellular component, molecular function, and biological process. Here, DEPs derived from the D/A and C/D comparisons were annotated via GO secondary classification (Figure 4A,B). COG/KOG functional classification was further performed to analyze the homologous functions of these DEPs, which reflected the metabolic and physiological characteristics of cells under different treatments. The DEPs from the D/A and C/D groups were subjected to COG/KOG functional classification (Figure 4C,D).

Fisher’s exact test was used for functional annotation and enrichment analysis of DEPs in the D/A and C/D groups. KEGG pathway enrichment was conducted to identify significantly enriched functional pathways of DEPs, with *p* < 0.05 defined as the threshold for significant enrichment. The top 20 most significantly enriched pathways were visualized in bubble plots (Figure 4E,F).

### 3.5. Cluster Analysis

**Figure 5:**
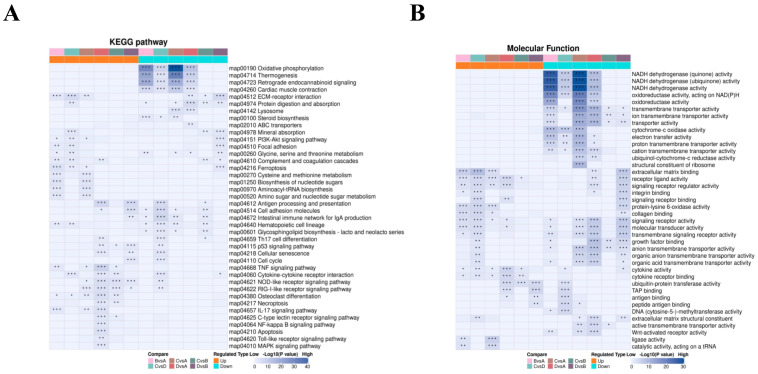
Cluster analysis of differentially expressed proteins. (**A**) Hierarchical clustering heatmap of KEGG pathways for differentially expressed proteins from four groups; (**B**) hierarchical clustering heatmap of molecular function terms for differentially expressed proteins from four groups. * *p* < 0.05, ** *p* < 0.01, and *** *p* < 0.001.

Based on the *p* values of Fisher’s exact test obtained from enrichment analysis, hierarchical clustering was performed to cluster the enriched functional terms across different comparison groups, and the results were visualized as heatmaps. In the heatmaps, the horizontal axis represents different comparison groups, and the vertical axis indicates functional terms enriched by differentially expressed proteins, including GO terms, KEGG pathways, and protein domains. The color gradient of each block corresponds to the enrichment significance. Blue indicates high enrichment significance, while blue-white represents low significance. Asterisks denote statistical significance: * *p* < 0.05, ** *p* < 0.01, and *** *p* < 0.001. Notably, combined with molecular function and KEGG pathway enrichment profiling (Figure 5A,B), our proteomic screening successfully identified the RIG-I-mediated innate immune signaling pathway and NF-κB inflammatory signaling pathway as the most significantly altered core pathways following JEV infection and magnolol intervention. These clustering results provided preliminary proteomic evidence for our subsequent mechanistic validation and defined the RIG-I/NF-κB axis as the key regulatory target of magnolol against JEV-induced inflammatory injury.

### 3.6. Validation of Differential Proteins in the RIG-I Signaling Pathway

**Figure 6:**
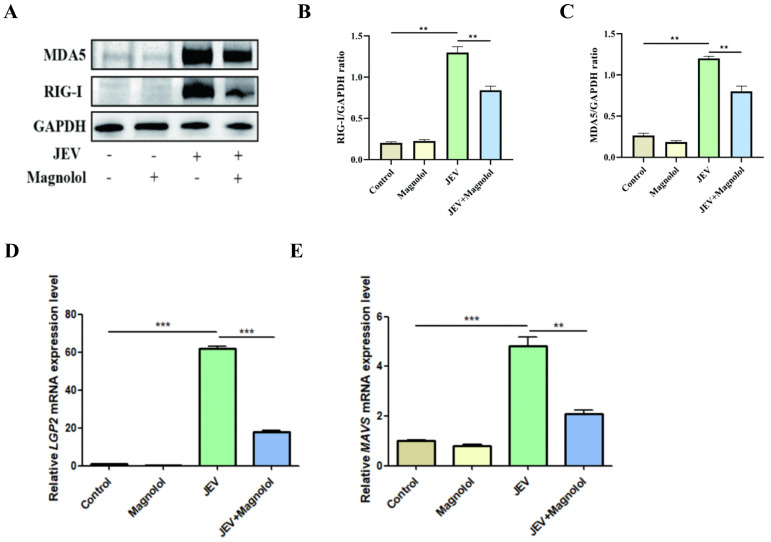
Effects of magnolol on the expression of RIG-I pathway-related proteins and genes in JEV-infected ST cells. (**A**–**C**) Relative protein expression levels of RIG-I and MDA5; (**D**,**E**) relative mRNA expression levels of *LGP2* and *MAVS*. ** *p* < 0.01, and *** *p* < 0.001.

Based on the quantitative proteomic results, key proteins involved in the RIG-I signaling pathway, including RIG-I, MDA5, LGP2, and MAVS, were validated at both transcriptional and protein levels. Compared with the control group, the protein levels of RIG-I and its positive regulator MDA5 were significantly upregulated in the JEV infection group. In contrast, magnolol treatment markedly reversed this upregulation and reduced the protein abundance of RIG-I and MDA5 in the JEV-stimulated cells (Figure 6A–C). In addition, JEV infection significantly increased the transcriptional levels of *LGP2* and the downstream adaptor *MAVS*, whereas magnolol intervention effectively decreased their transcription in the JEV-treated group (Figure 6D,E). Collectively, these results demonstrated that JEV infection activates the RIG-I signaling pathway, and 24 h treatment with 16 μg/mL magnolol extremely significantly inhibits the activation of this pathway.

### 3.7. Verification of the NF-κB Signaling Pathway

**Figure 7:**
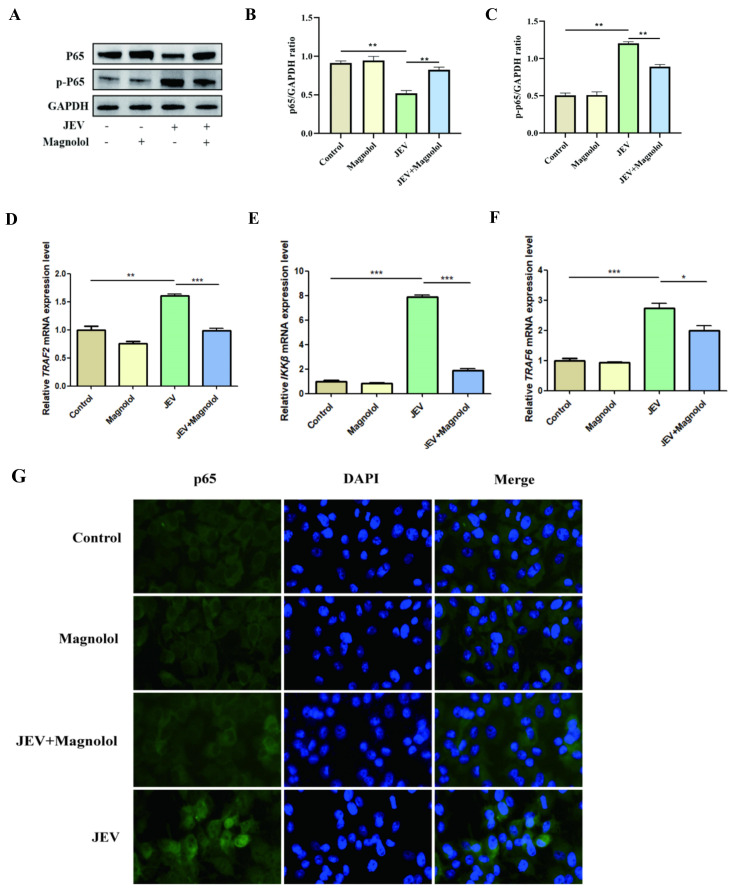
Effects of magnolol on the expression of NF-κB pathway-related proteins and genes in JEV-infected ST cells. (**A**–**C**) Relative protein expression of total p65 and phosphorylated p65 (pp65); (**D**–**F**) relative mRNA expression of TRAF2, IKKβ and TRAF6; (**G**) effect of magnolol on JEV-triggered nuclear translocation of p65. * *p* < 0.05, ** *p* < 0.01, and *** *p* < 0.001.

Proteomic analysis confirmed that magnolol inhibited the JEV-induced activation of the RIG-I signaling pathway. Accordingly, we further verified the activity of the NF-κB signaling pathway, a downstream cascade of RIG-I. As shown in (Figure 7A–C), JEV infection significantly upregulated the phosphorylation level of p65, whereas magnolol treatment markedly reversed this increase. Consistent with this result, JEV infection extremely significantly increased the transcript levels of NF-κB positive regulatory genes including *IKKβ*, *TRAF2* and *TRAF6*, while magnolol treatment effectively downregulated the expression of these genes (Figure 7D,F). These results indicated that JEV infection activates the NF-κB signaling pathway, and magnolol treatment potently alleviates this activation.

Phosphorylation and nuclear translocation of p65 is a hallmark of NF-κB activation. To further intuitively verify the inhibitory effect of magnolol on p65 activation, the nuclear translocation of p65 was observed by fluorescence microscopy (Figure 7G). In this assay, green fluorescence represented p65 protein, and blue fluorescence-stained nuclei with DAPI. The transcriptional function of NF-κB is triggered once p65 translocates into the nucleus. In the control group, p65 was evenly distributed in the cytoplasm. After JEV infection, p65 was obviously enriched in the nucleus. Notably, magnolol treatment remarkably reduced JEV-induced p65 nuclear translocation. Collectively, these findings further demonstrated that magnolol could suppress JEV-triggered NF-κB signaling activation.

## 4. Discussion

As a zoonotic single-stranded RNA virus, JEV not only triggers severe central nervous system lesions in humans [28], but also serves as a major pathogen that drastically impairs reproductive performance in breeding boars. After viral nucleic acids are recognized by host cells, a severe local inflammatory storm is initiated [29]. Clinically, infected boars present testicular swelling, asthenospermia and oligozoospermia. In severe cases, boars suffer permanent loss of breeding capacity, leading to enormous economic losses in intensive pig farms. Vaccination remains the primary strategy for JEV prevention at present, yet it has prominent drawbacks including limited immune duration and susceptible immune blank periods in piglets and replacement boars. In addition, rapid mosquito-borne transmission during epidemic seasons makes complete infection blockade difficult. Synthetic glucocorticoids, commonly used to relieve inflammatory symptoms, can suppress normal host immunity, leave drug residues and disrupt reproductive endocrine function. Therefore, exploring safe, low-toxicity and residue-free natural bioactive compounds to alleviate JEV-induced testicular inflammatory injury has become a hot research topic in the prevention and control of swine reproductive infectious diseases.

Magnolol, the core bioactive polyphenol extracted from the traditional medicinal herb Magnolia officinalis, has been well documented to exert broad-spectrum anti-inflammatory, antioxidant and antiviral effects in recent pharmacological studies [30,31]. In terms of antiviral activity, multiple in vitro cellular assays have confirmed that magnolol interferes with the infection process of diverse positive-sense single-stranded RNA viruses such as dengue virus, porcine reproductive and respiratory syndrome virus and influenza A virus. For its anti-inflammatory function, most existing research relies on macrophage and neuronal cell models, proving that magnolol reduces pro-inflammatory cytokine secretion by inhibiting multiple innate immune inflammatory cascades. Nevertheless, studies focusing on magnolol against JEV infection are scarce, and few reports have explored its protective mechanisms in porcine testicular cell models with reproductive damage phenotypes. The present study established an in vitro JEV infection model using swine testicular (ST) cells and systematically dissected the protective effects of magnolol against JEV-triggered inflammatory injury, filling the research gap in this field.

In this work, 4D-FastDIA quantitative proteomics was applied to globally profile dynamic proteomic alterations in ST cells upon JEV infection and magnolol treatment, providing unbiased omics evidence for downstream signaling screening. Rigorous quality control validated high reproducibility and reliability of the proteomic dataset. Functional enrichment of differentially expressed proteins was consistent with previous research regarding host responses to JEV: differentially expressed proteins induced by JEV were predominantly enriched in pathways governing innate immune recognition and NF-κB-mediated inflammatory responses. This finding indicates that viral invasion immediately activates host immune and inflammatory programs, forming the molecular basis of cellular inflammatory burst. Comparison between the JEV-infected group and magnolol co-treatment group revealed that magnolol extensively reversed JEV-triggered abnormal expression of immune-related proteins. At the whole-proteome level, magnolol remodels dysregulated host innate immune responses and prevents sustained amplification of inflammatory signals, offering solid omics support for subsequent targeted verification of the RIG-I/NF-κB signaling axis.

Retinoic acid-inducible gene I (RIG-I) acts as the principal cytoplasmic pattern recognition receptor sensing viral double-stranded RNA [32], serving as the initial signal switch for host cells to detect JEV invasion. Excessive activation of this pathway is a central contributor to inflammatory damage induced by RNA viruses. The previous literature has reported that JEV infection significantly upregulates RIG-I and MDA5 expression in neuronal and renal cells. After recognizing viral nucleic acids, RIG-I and MDA5 bind to the downstream adaptor protein MAVS to amplify immune signals stepwise. Consistent with these findings, our mRNA and protein dual-level validation illustrated that the expression of RIG-I, MDA5 protein, LGP2 and MAVS transcripts was extremely elevated in the single JEV infection group, fully verifying that JEV potently activates RIG-I signaling in porcine testicular cells. Gradient magnolol intervention suppressed the overexpression of core RIG-I pathway molecules in a dose-dependent manner, with the optimal inhibitory effect observed at 16 μg/mL. This outcome demonstrates that magnolol targets excessive RIG-I cascade activation and blocks upstream initiation and transduction of inflammatory signals. Such a regulatory mechanism resembles other natural polyphenols including baicalein and quercetin that modulate RIG-I pathways [33,34], whereas our research based on reproductive cell models better mimics the pathological background of JEV-induced infertility in boars under practical pig production conditions.

The NF-κB pathway functions as a critical downstream effector of RIG-I that mediates massive transcription of pro-inflammatory cytokines and acts as the core amplifier of inflammatory cascades. Under resting conditions, p65 resides in the cytoplasm without transcriptional activity [35]. Once upstream RIG-I signaling activates TRAF2 and TRAF6 and upregulates IKKβ, the IKK complex mediates p65 phosphorylation. Phosphorylated p65 translocates into the nucleus, binds to promoters of target genes, and drives abundant synthesis and secretion of pro-inflammatory mediators such as TNF-α and IL-1β [14]. In vivo pathological experiments in boars have proven that persistent NF-κB activation and massive p65 nuclear translocation in JEV-infected testicular tissues are direct inducers of testicular edema and cellular apoptosis. Our results suggest that magnolol simultaneously blocks upstream RIG-I viral recognition signals and downstream NF-κB inflammatory transcriptional programs, completely cutting off the RIG-I/NF-κB inflammatory signaling axis rather than merely regulating a single target, thus exerting comprehensive anti-inflammatory protective effects.

Collectively, all experimental data in this study confirm that JEV induces inflammatory injury in ST cells by hyperactivating the RIG-I/NF-κB signaling axis, while magnolol alleviates viral-mediated cellular inflammation by inhibiting overactivation of this cascade and downregulating multiple pro-inflammatory cytokines. Testicular inflammatory responses are driven by immune cell infiltration and massive release of pro-inflammatory cytokines and reactive oxygen species (ROS), which disrupt the blood–testis barrier (BTB), trigger extensive apoptosis and shedding of germ cells, and markedly impair the function of Sertoli cells, thereby severely disturbing spermatogenesis and steroid synthesis. After inflammation subsides, immune infiltration gradually diminishes and oxidative stress is alleviated. Surviving Sertoli cells reconstruct tight junctions to restore BTB structural integrity, while residual spermatogonial stem cells (SSCs) resume proliferation and differentiation, ultimately recovering spermatogenesis and testosterone production. This study provides sufficient in vitro experimental evidence for developing natural botanical agents to mitigate JEV-induced cellular inflammatory injury. However, several limitations remain to be addressed. First, all mechanistic verifications were performed exclusively using immortalized ST cells. Compared with primary porcine testicular cells and intact testicular tissues in vivo, the immortalized cell line possesses distinct biological characteristics and cannot fully recapitulate the complex physiological microenvironment and immune regulatory network of porcine testis. Accordingly, the current findings only provide reliable cellular-level mechanistic evidence for the anti-inflammatory and protective effects of magnolol against JEV infection, and cannot completely represent the therapeutic efficacy in living animals. To further verify the translational potential of magnolol in veterinary clinical practice, subsequent studies will be necessary to isolate primary porcine testicular cells and establish in vivo JEV-infected animal models, so as to validate the protective effect and molecular mechanism of magnolol at the tissue and individual levels, and further promote the practical application of magnolol in the prevention and treatment of JEV-induced porcine reproductive disorders. Second, this research only focused on magnolol’s anti-inflammatory capacity without distinguishing its direct antiviral activity against JEV replication or its isolated inhibitory effect on inflammatory pathways. Subsequent TCID_50_ assays should be performed to detect whether magnolol directly suppresses JEV proliferation. Third, only one magnolol concentration gradient with short-term intervention was tested in this study. Long-term toxicity assessment and determination of safe cellular concentration ranges are absent, and additional safety evaluation trials are needed before its application as feed additives or veterinary drugs.

In summary, the present study clarifies the molecular mechanism by which magnolol alleviates JEV-induced inflammatory damage in porcine testicular cells and identifies the RIG-I/NF-κB signaling axis as its core target. It provides theoretical support and experimental references for developing green, residue-free natural anti-inflammatory additives to reduce elimination losses of breeding boars caused by JEV outbreaks on pig farms.

## 5. Conclusions

This study explored the protective mechanism of magnolol against JEV-induced inflammation in ST cells. JEV infection activated the RIG-I/NF-κB signaling axis, promoted p65 phosphorylation and nuclear translocation, and induced excessive expression of pro-inflammatory cytokines, resulting in severe cellular inflammatory damage. In contrast, magnolol effectively inhibited the overactivation of RIG-I/NF-κB signaling, blocked p65 nuclear translocation, and reversed JEV-triggered inflammatory responses. Collectively, magnolol alleviates JEV-mediated cell injury by modulating the RIG-I/NF-κB pathway. This work provides solid experimental support for the application of magnolol as a natural agent against JEV.

## Figures and Tables

**Table 1:** Primer sequences used for quantitative real-time PCR.

Gene	PCR. Gene Sequence
β-actin F	5′-CTTCCTGGGCATGGAGTCC-3′
β-actin R	5′-GGCGCGATGATCTTGATCTTC-3′
TNF-α F	5′-GCCCAAGGACTCAGATCATC-3′
TNF-α R	5′-GGCATTGGCATACCCACTCT-3′
CCL-5 F	5′-ACACCCTGCTGTTTTTCCTACCT-3′
CCL-5 R	5′-AGACGACTGCTGCCATGGA-3′
IL-6 F	5′-CTGCTTCTGGTGATGGCTACTG-3′
IL-6 R	5′-GGCATCACCTTTGGCATCTT-3′
IL-1β F	5′-ACAAAAGCCCGTCTTCCTG-3′
IL-1β R	5′-ATGTGGACCTCTGGGTATGG-3′
IL-8-F	5′-AGTTTTCCTGCTTTCTGCAGCT-3′
IL-8-R	5′-TGGCATCGAAGTTCTGCACT-3′
LGP2-F	5′-CCACCAAGAACCCAGATCCTA-3′
LGP2-R	5′-GACCCTTGAACTCCCCTGAAG-3′
TRAF2-F	5′-CCACCGCTACTGCTCCTACTGC-3′
TRAF2-R	5′-CGCCTTCTTCATAAATGCCCTC-3′
IKKβ-F	5′-AGAGGATCTTCTGCGAGTA-3′
IKKβ-R	5′-CTTTGGGTGCGTAACTG-3′
TRAF6-F	5′-CAAGAGAATACCCAGTCGCACA-3′
TRAF6-R	5′-ATCCGAGACAAAGGGGAAGAA-3′

## Data Availability

All data generated in this study are available within the manuscript.

## Abbreviations

The following abbreviations are used in this manuscript:

JEV	Japanese encephalitis virus
PRRs	Pattern recognition receptors
TLRs	Toll-like receptors
PAMPs	Pathogen-associated molecular patterns
CCK	Cell counting kit
RSD	Relative standard deviation
PCA	Principal component analysis
GO	Gene ontology
KEGG	Kyoto Encyclopedia of Genes and Genomes
CC	Cellular component
MF	Molecular function
IL-6	Interleukin-6
TRAF2	Tumor Necrosis Factor Receptor-Associated Factor 2
IKKβ	Inhibitor of Nuclear Factor-kappa B Kinase Beta
DMSO	Dimethyl Sulfoxide
